# Presence of Adenomyosis Impairs Clinical Outcomes in Women Undergoing Frozen Embryo Transfer: A Retrospective Cohort Study

**DOI:** 10.3390/jcm12186058

**Published:** 2023-09-19

**Authors:** Noémie Sachs-Guedj, Buenaventura Coroleu, María Ángela Pascual, Ignacio Rodríguez, Nikolaos P. Polyzos

**Affiliations:** 1 Department of Obstetrics, Gynecology and Reproduction, Dexeus University Hospital, 08028 Barcelona, Spain; noesac@dexeus.com (N.S.-G.); vencor@dexeus.com (B.C.); marpas@dexeus.com (M.Á.P.); nacrod@dexeus.com (I.R.); 2 Department of Pediatrics, Obstetrics and Gynecology, Faculty of Medicine, Autonomous University of Barcelona, 08193 Barcelona, Spain; 3 Faculty of Medicine and Health Sciences, Ghent University (UZ Gent), 9000 Gent, Belgium

**Keywords:** adenomyosis, frozen embryo transfer, GnRH agonist, MUSA criteria

## Abstract

(1) Background: The presence of adenomyosis among pregnant patients has been associated with a higher incidence of miscarriage and pregnancy complications. Although the role of adenomyosis in women undergoing in vitro fertilization (IVF) was investigated in several studies and demonstrated a potentially detrimental effect on live birth rates following IVF, most of them were small studies in which the adenomyosis diagnosis was not confirmed based on solid ultrasonographic criteria. (2) Methods: 3503 patients undergoing their first blastocyst frozen transfer through a hormonal replacement (HRT) FET cycle. Among them, 140 women had a confirmed diagnosis of adenomyosis based on the MUSA criteria. (3) Results: Adenomyosis patients were more likely to proceed with deferred FET compared with no-adenomyosis women (*p* = 0.002) and were significantly more likely to be treated with GnRH agonist pre-treatment (2 months) (*p* < 0.001). The presence of adenomyosis significantly decreased the clinical pregnancy rates (aOR 0.62, 95% CI: 0.39–0.98, *p* = 0.040) and live birth rates (aOR 0.46, 95% CI: 0.27–0.75, *p* = 0.003) and significantly increased the miscarriage rates (aOR 2.13, 95% CI: 0.98–4.37, *p* = 0.045). Multivariable logistic regression adjusting for age, autologous or donor oocytes, PGT-A, deferred FET, serum progesterone levels the day before FET, GnRH agonist pre-treatment, number of embryos transferred, and adenomyosis demonstrated that the use of the GnRH agonist protocol did not decrease or increase the miscarriage rate, clinical pregnancy rate, or live birth rate. (4) Conclusions: The presence of adenomyosis had a significant negative impact on the clinical outcomes of patients undergoing FET and was associated with higher miscarriage, lower clinical pregnancy, and live birth rates. GnRH agonist pre-treatment does not appear to improve clinical outcomes.

## 1. Introduction

Adenomyosis is a gynecologic disease characterized by ectopic endometrial glands and stroma within the uterine myometrial layer [1]. The presence of adenomyosis can lead to various changes in the endometrium, inflammation, abnormal uterine contractility, and infertility. Clinical presentations of adenomyosis vary, with abnormal uterine bleeding and dysmenorrhea being the most common, although some patients may remain asymptomatic.

The impact of adenomyosis on the success of in vitro fertilization (IVF) has been a subject of debate [1]. A recent meta-analysis suggested that adenomyosis might have a negative impact on the clinical pregnancy rate [2], miscarriage rate [2,3], and live birth rate [2] in IVF cycles. However, it is important to note that not all studies shared the same perspective as the prospective study conducted by Neal et al., 2020 [4]. The conflicting results are attributed to the high degree of heterogeneity among studies and, in particular, in the diagnosis of adenomyosis, which is not always confirmed based on solid ultrasonographic criteria. Since 2015, the MUSA consensus was established to diagnose adenomyosis. This consensus was developed by different experts to facilitate the diagnostic process by defining both direct and indirect signs of adenomyosis, thereby unifying the diagnostic approach. The MUSA criteria provide a standardized framework for describing and reporting the sonographic features of the myometrium [5]. The ongoing revision of the MUSA criteria serves to continually enhance the accuracy and quality of adenomyosis diagnosis [6].

Given the lack of strong evidence and the conflicting findings in the existing literature, we conducted a retrospective cohort study with strict inclusion criteria at Dexeus University Hospital in order to investigate the actual impact of adenomyosis on IVF clinical outcomes. By carefully examining a well-defined patient population and by using the MUSA criteria, our aim was to ensure consistency and reliability in the definition of adenomyosis within our study population. The first objective was to provide clearer insights into the relationship between adenomyosis and IVF success rates.

Up to date, no consensus on the optimal ET technique exists [7]. Embryo transfer (ET) is one of the main components of IVF success [8] and can be a very challenging process, especially for patients with uterine diseases, including adenomyosis [9].

Treatment with suppressive hormonal therapies, which include the administration of oral contraceptive pills, high-dose progestins, selective estrogen receptor modulators, selective progesterone receptor modulators, the levonorgestrel-releasing intrauterine device, aromatase inhibitors, danazol, and gonadotropin receptor hormone agonists (GnRH-a), can provide temporary relief by inducing the regression of adenomyosis and alleviating associated symptoms [10]. GnRH-a, which is a treatment largely used due to the hypoestrogenic effect and antiproliferative effect within the myometrium, was shown to improve spontaneous pregnancies for patients suffering from adenomyosis by restoring endometrial receptivity. Nevertheless, the available literature for patients undergoing a long agonist protocol or using GnRH-a pre-treatment before IVF is limited and highly heterogeneous. Recent data suggested that GnRH-a may not have a beneficial effect on IVF outcomes in women with adenomyosis, as suggested by studies conducted by Li et al., 2021 and Cozzolino et al., 2022 [2,11].

Considering the controversial role of GnRH-a in patients with adenomyosis, the second objective was to analyze whether GnRH-a downregulation could be an effective intervention in endometrial preparation protocols with FET based on hormone replacement therapy (HRT) cycles for patients with adenomyosis.

## 2. Materials and Methods

This retrospective cohort study was conducted at the Dexeus University Hospital, which is a university-affiliated fertility center. We analyzed the data from the hospital electronic database for patients undergoing a first HRT cycle of blastocyst frozen transfer between January 2017 and December 2021. The decision to include only the HRT cycle was made considering that adenomyosis is a hormonally responsive disease, where the use of an HRT cycle could reduce inflammation associated with the condition and control estradiol levels [12].

In order to ensure a homogeneous group of patients with adenomyosis, we employed rigorous criteria for inclusion. We included donor and autologous oocytes with or without preimplantation genetic testing for aneuploidy (PGT-a). The exclusion criteria were fresh embryo transfer, natural or modified natural cycle preparation in frozen blastocyst transfer, hydrosalpinx, endometriosis, or any intracavity disease (e.g., polyp or submucosal myoma). In order to minimize confounding factors, we made the decision to entirely exclude patients with endometriosis from our study. We were particularly concerned about potential biases due to the close relationship between endometriosis and adenomyosis. Although both disorders involve the presence of endometrioid tissue in ectopic locations distant from the endometrium [13], each disease can manifest independently. Moreover, endometriosis is a known confounding factor and an additional cause of infertility [14].

In total, 3503 patients met the inclusion criteria. Of those patients, 140 patients were diagnosed with adenomyosis using the Morphological Uterus Sonographic Assessment (MUSA) criteria. The diagnosis of adenomyosis was exclusively made by expert gynecologic imaging specialists from the hospital imaging department.

### 2.1. Procedure

For the HRT cycle regimen, the endometrium prepared with oral estradiol valerate at a dose of 6 mg daily was started on days 1–3 of the menstrual cycle. Endometrial thickness was monitored by transvaginal ultrasound after 10–12 days of medication. Thereafter, the dose of estradiol valerate was adjusted according to the endometrial thickness (8 mg/d maximally or estradiol patch supplementation 150 mcg/72 h, in addition to initial treatment if the endometrial thickness was less than 7 mm). Micronized vaginal progesterone (MVP) at 600 mg/day was added when the endometrial thickness reached 7 mm or more. All patients underwent a serum progesterone (P) measurement on the day before the embryo transfer. Patients with P < 10.6 ng/mL received a supplement of progesterone (subcutaneous injection of 25 mg), while patients with P ≥ 10.6 ng/mL maintained the previous luteal phase support (LPS) protocol. Frozen embryo transfer (FET) was carried out 6 days after the start of the LPS. Estradiol valerate at the dose for endometrial preparation was continued until the day of the serum hCG test, which was 10 days after the embryo transfer. If pregnancy was achieved, estradiol valerate and MVP were stopped at 10 weeks of amenorrhea if < 45 years old or at 12 weeks of amenorrhea if > 45 years old.

When decided by the gynecologist, the administration of GnRH-a injection at a dose of 3.75 mg for 2 months before the HRT protocol was performed. The 2-month doses were decided according to the hospital protocol.

### 2.2. Outcome Measures

The primary outcome of this study was the live birth rate. The secondary outcomes included the clinical pregnancy rate, miscarriage rate, and the GnRH-a pre-treatment administration in the adenomyosis patients’ group. Live birth was defined as the number of deliveries that resulted in at least one live-born baby. Clinical pregnancy was defined as a pregnancy diagnosed by ultrasonographic intrauterine visualization of one or more gestational sacs 2 to 3 weeks after embryo transfer. A miscarriage was defined as a fetal loss before the 20th week of gestation.

### 2.3. Statistical Analysis

Continuous variables were described using means and standard deviations, while the categorical variables were described using frequencies and percentages. To compare the distributions between groups, the *t*-test for continuous variables or Fisher’s exact test and the chi-squared test for categorical variables were used.

Multivariable logistic regression adjusted for age, autologous or donor oocytes, PGT-a, freeze-all protocol, serum progesterone levels the day before FET, GnRH-a pre-treatment, N of embryos transferred, and adenomyosis was used to analyze the relationship between adenomyosis and the clinical outcomes. All tests were bilateral with a significance level set at 5%. The statistical analyses were performed in R software [15].

## 3. Results

In total, 3503 women were included in this study. Of these, 3363 were in the control group and 140 were in the adenomyosis group.

### 3.1. Baseline Characteristics

The baseline values of the patients are presented in Table 1.

The baseline characteristics of our patients were notably homogeneous in both groups. There was no significant difference in height, weight, or body mass index (BMI) (164.09 ± 6.28 vs. 163.81 ± 5.95, *p* = 0.61; 63.95 ± 12.55 vs. 64.67 ± 13.32, *p* = 0.56; 23.72 ± 4.51 kg/m^2^ vs. 23.86 ± 4.31 kg/m^2^, *p* = 0.72, respectively) between the two groups. The percentages of types of oocytes transferred (embryo donation, oocyte donation, autologous IVF), nulliparous women, smoker, and PGT-a, and good-quality embryos (GQEs) (A or B, as per the ASEBIR’s morphological scoring system) were comparable between the two groups (7.29% vs. 7.14%, 38.24% vs. 42.14%, 54.48% vs. 50.71%, *p* = 0.64; 96.49% vs. 95.71%, *p* = 0.64; 18.23% vs. 16.43%, *p* = 0.59; 23.85% vs. 22.86%, *p* = 0.79; 0 GQE 32.71% vs. 35.71%, 1 GQE 63.84% vs. 62.86%, 2 GQE 3.45% vs. 1.43%, *p* = 0.39, respectively). The endometrial thickness and the mean progesterone and estradiol levels the day before the blastocyst transfer were not significantly different between the two groups (10.29 ± 2.03 vs. 10.27 ± 2.61, *p* = 0.94; 12.53 ng/mL ± 6.27 vs. 12.17 ng/mL ± 4.20, *p* = 0.45; 224.28 pg/mL ± 118.14 vs. 203.64 pg/mL ± 112.52, *p* = 0.10, respectively). The number of transferred embryos was generally no more than one and did not differ between the two groups (1.07 ± 0.21 vs. 1.05 ± 0.27, *p* = 0.12).

However, we observed a significant difference in the age of women at the time of transfer, with those in the adenomyosis group being slightly older (39.84 ± 5.23 vs. 40.75 ± 4.71, *p* = 0.028). When considering the age of the oocytes at the time of blastocyst transfer, no significant differences were observed between the two groups (31.83 ± 6.54 years vs. 31.56 ± 6.98 years, *p* = 0.69).

Another notable disparity between the groups was the higher frequency of freeze-all protocols in the adenomyosis group (63.60% vs. 76.43%, *p* = 0.002).

Furthermore, we observed a higher likelihood of GnRH-a pre-treatment in patients with adenomyosis compared to the control group (9.69% vs. 20%, *p* = <0.001).

### 3.2. Adenomyosis vs. No Adenomyosis and Reproductive Outcomes

#### 3.2.1. Unadjusted Results

**Figure 1:**
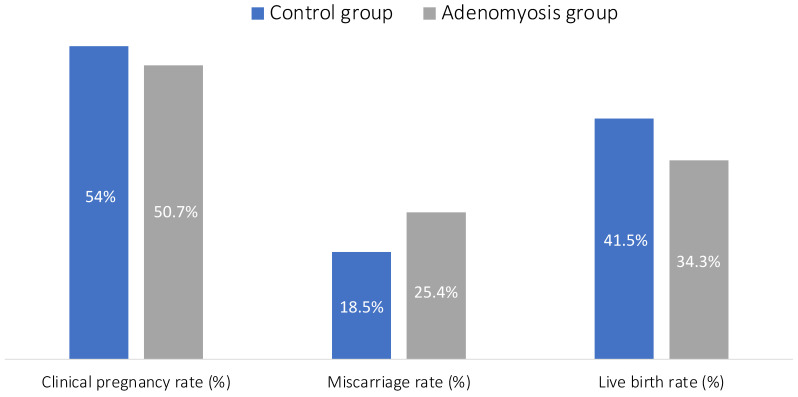
Adenomyosis and reproductive outcomes unadjusted results.

Figure 1 presents a comparison of reproductive outcomes between the two groups.

The analysis of unadjusted results failed to show any significant difference between women with adenomyosis vs. the control group, in the clinical pregnancy rate (53.97% vs. 50.71%, *p* = 0.45), the miscarriage rate (18.46% vs. 25.35%, *p* = 0.14), and the live birth rate (41.54% vs. 34.29%, *p* = 0.09).

#### 3.2.2. Adjusted Multivariable Logistic Regressions

**Figure 2:**
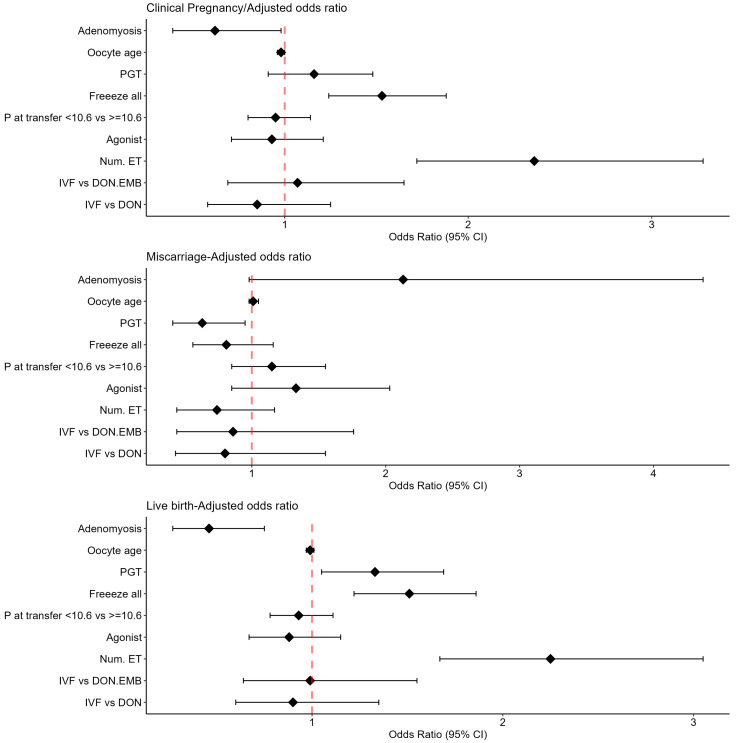
Clinical pregnancy, miscarriage, and live birth adjusted odds ratios.

When performing the multivariable logistic regressions (Figure 2), we observed that the use of a freeze-all protocol and the number of embryos transferred showed a positive association with the clinical pregnancy rate (aOR 1.53, 95% CI 1.24 to 1.88; aOR 2.36, 95% CI 1.72 to 3.28, respectively), while adenomyosis exhibited a negative association with the clinical pregnancy rate (aOR 0.62, 95% CI 0.39 to 0.98) (Table 2). Regarding the miscarriage rate, PGT-a was associated with a decrease in the miscarriage rate (aOR 0.63, 95% CI 0.41 to 0.95), while adenomyosis exhibited a positive association with the miscarriage rate (aOR 2.13, 95% CI 0.98 to 4.37) (Table 3). Concerning the live birth rate, PGT-a, the use of a freeze-all protocol, and the number of embryos transferred showed positive associations with the live birth rate (aOR 1.33, 95% CI 1.05, 1.69; aOR 1.51, 95% CI 1.22 to 1.86; aOR 2.25, 95% CI 1.67 to 3.05, respectively), while adenomyosis exhibited a negative association with the live birth rate (aOR 0.46, 95% CI 0.27 to 0.75) (Table 4).

### 3.3. GnRH Agonist vs. No Treatment in Adenomyosis Group

In the adenomyosis group, the multivariable logistic regression analysis results (Table 5, Table 6 and Table 7) show that the administration of GnRH agonist pre-treatment for two months did not improve the clinical birth rate or live birth rate, nor did it change the miscarriage rate.

## 4. Discussion

Our study provides evidence showing the detrimental impact of adenomyosis on pregnancy outcomes following FET for ART. After adjustment for age, autologous or donor oocytes, PGT-a, freeze-all protocol, serum progesterone levels the day before FET, GnRH-a pre-treatment, N of embryos transferred, and adenomyosis, we found that adenomyosis impaired the clinical pregnancy, live birth, and miscarriage rates. Administration of 2 months GnRH-a prior to FET did not improve the clinical pregnancy, miscarriage, or live birth rates among the study population.

Upon analyzing the results of our multivariable logistic regression analysis, we observed a positive association between the implementation of a freeze-all protocol and the clinical pregnancy rate, as well as the live birth rate. This finding can be attributed to the fact that the freeze-all protocol represents the first cycle of embryo transfer for these patients, unlike those who had previously undergone a fresh blastocyst cycle prior to participating in the study. Logically, the number of embryos transferred demonstrated a positive impact on both the clinical pregnancy and live birth rates. This can be attributed to the increased likelihood of successful implantation and development when multiple embryos are transferred [16]. Additionally, PGT-a exhibited a positive impact on reducing miscarriage rates and improving live birth rates. These findings align with the notion that PGT-a helps to select genetically normal embryos, thereby may increase the chances of a successful pregnancy and live birth per embryo transfer in older age patients [17].

Our study results are consistent with a recent meta-analysis of 22 studies conducted by Cozzolino et al., which demonstrated a detrimental impact of adenomyosis on pregnancy outcomes for patients undergoing ART [2]. However, it is important to note that this meta-analysis combined studies that performed different protocols for embryo transfer, included patients with endometriosis, and used various criteria to diagnose adenomyosis. One of the studies included in this meta-analysis was the only prospective study that conducted a three-dimensional (3D) ultrasound (US) assessment of adenomyosis to evaluate the presence of sonographic features based on the MUSA criteria [4]. The 3D US was performed by the attending physician in the office and reviewed independently by five reproductive endocrinologists, each with experience in conducting a minimum of 1000 USs in the preceding year. Among the 638 patients who underwent a thawed euploid blastocyst transfer, the prevalence of adenomyosis was 15.3% and no significant difference was observed in the rates of clinical pregnancy, miscarriage, or live birth. It is worth noting that the disparity in inter-rater agreement in 3D US, which was found to be poor, could explain the difference with our study results. In our research, the ultrasounds were exclusively performed by expert gynecologic imaging specialists, who performed the USs in real-time, thus providing more comprehensive information than a professional who would review the US exam of another performer without performing the US [18]. This difference in examination methodology may explain the inconsistency in our results compared with Neal’s study.

Since the publication of Cozzolino’s meta-analysis, several retrospective studies have emerged in the field. One study conducted in China published in 2021 focused on infertile patients undergoing IVF and frozen–thawed embryo transfer. The study excluded patients with endometriosis and evaluated various transfer protocols, including a natural cycle, an HRT cycle, and downregulation followed by an HRT cycle. The conclusions drawn from this study align with our findings concerning the miscarriage rate and the live birth rate. However, they did not observe a significant impact of adenomyosis on the embryo implantation rate or clinical pregnancy rate. This result can be attributed to the significantly higher rate of high-quality embryos in their adenomyosis group than in their control group [19].

One limitation of our study was its retrospective nature. Additionally, it should be noted that the impact of adenomyosis on pregnancy outcomes might vary depending on the severity of uterine involvement and subtype, which were not considered in this study. Indeed, while the sample size of patients with adenomyosis who met strict inclusion criteria was a strength of our study, the overall number of such patients was not sufficient for a comprehensive subgroup analysis of pregnancy outcomes.

In the literature, a recent retrospective study reported that patients with focal adenomyosis or with tubal infertility may have better reproduction outcomes compared with patients with diffuse adenomyosis following an embryo transfer with an ultra-long GnRH agonist protocol [20]. Similarly, a recent meta-analysis reported that diffuse adenomyosis had an adverse impact on in vitro fertilization outcomes, resulting in lower live birth and clinical pregnancy rates, while the miscarriage rates remained unchanged [21]. Furthermore, other data show that an increase in uterine volume can negatively affect reproductive outcomes in patients with adenomyosis. A 2023 retrospective study conducted in China that included patients from 2009 until 2019 highlighted that patients undergoing their first FET with a uterine volume of more than 130 cm^3^ had a higher rate of miscarriage [22]. Another retrospective study, conducted by Li et al., shared the same conclusion and suggested that a uterine volume of more than 98.81 cm^3^ might lead to a lower live birth rate due to a higher incidence of miscarriage [23]. On the other hand, Wang et al. reported that symptomatic adenomyosis was found to affect live birth, clinical pregnancy, and miscarriage rates, while asymptomatic adenomyosis may not have a significant impact on pregnancy outcomes [21]. However, to date, no studies have investigated the influence of different subtypes of adenomyosis diagnosed using the MUSA criteria on reproductive outcomes. This is why the association between clinical subtypes of adenomyosis and reproductive outcomes remains uncertain, warranting further exploration.

Regarding our second outcome, the lack of any effect of the 2-month GnRH-a administration in patients with adenomyosis in terms of clinical pregnancy, miscarriage, or live birth could be related to several factors.

First, as this was not our study’s primary outcome, it is possible that the sample size of 140 patients was not large enough to detect statistically significant differences. Nonetheless, our results align with those of one prospective study conducted on 100 patients undergoing an HRT cycle, where the treatment group received GnRH-a the month before the FET [24]. Other recent retrospective studies also shared the same conclusion [11,25]. In Chen et al.’s study, patients received up to 3 months of GnRH-a administration before starting the HRT–FET protocol. The live birth rate was higher in the non-pre-treatment group than in the GnRH-a pre-treatment [25]. However, patients with endometriosis were included, which can be a great risk of bias. Li et al.’s study analyzed the cycle of 341 patients with adenomyosis and excluded the presence of endometriosis [11]. In this study, 89.34% of the patients experienced one or two GnRH-a administrations. The results showed no significant difference in the clinical pregnancy, miscarriage, or live birth rates.

Second, the duration of GnRH-a administration prior to the FET might be insufficient, and patients could potentially require a longer GnRH-a administration to experience any benefit based on the severity of adenomyosis, which was not considered in the study. While GnRH-a has been known to suppress the severity of adenomyosis lesions, the optimal duration of administration and its role in pregnancy outcomes have been the subject of ongoing debate. In some cases, severe adenomyosis can continue to manifest a hyperestrogenism status due to local production from the lesions, even after long-term treatment with GnRH-a.

Lastly, recent studies brought into question the efficacy of GnRH-a in consistently providing sufficient suppression of the axis to significantly impact adenomyosis [11,26], as the increased estrogen levels in the endometrium are attributed to the overexpression of aromatase P450 [27], a decrease in the conversion of estradiol into estrone, and the secretion of adenomyotic tissue itself.

Recent case studies suggested that combining Letrozole administration in hyperestrogenic patients may lead to improved outcomes [26]. To enhance the success of embryo transfer and overall in vitro fertilization outcomes, alternative treatment protocols have been implemented. Dianogest and levonorgestrel intrauterine systems have shown the potential to benefit the pregnancy outcomes of infertile patients with adenomyosis when undergoing FET [28,29]. Future prospective studies are needed to establish a consensus on the optimal embryo transfer technique for these patients.

## 5. Conclusions

Our study findings indicate that the presence of adenomyosis is associated with higher miscarriage and lower clinical pregnancy and live birth rates during artificial cycle frozen blastocyst transfer. Considering the adenomyosis group sample size, there is a need for larger studies encompassing specific subgroups. Interestingly, our analysis revealed that adenomyosis patients, whether receiving GnRH agonist treatment (2 months) based on the HRT cycle or not, exhibited similar reproductive outcomes. This suggests that the GnRH agonist treatment might not adequately suppress inflammation and estrogen production by the adenomyotic lesion. Finding alternative treatment strategies is necessary, and future prospective studies are needed to establish a consensus on the optimal embryo transfer technique for these patients.

## Figures and Tables

**Table 1:** Baseline Characteristics.

Patient Characteristics	Adenomyosis		*p*-Value ^2^
	No, N = 3363 ^1^	Yes, N = 140 ^1^	
FET (3503)			0.64
Embryo donation	245 (7.29%)	10 (7.14%)	
Oocyte donation	1286 (38.24%)	59 (42.14%)	
Autologous oocyte	1832 (54.48%)	71 (50.71%)	
Parity (3503)			0.64
Multiparous	118 (3.51%)	6 (4.29%)	
Nulliparous	3245 (96.49%)	134 (95.71%)	
Smoking status (3503)	613 (18.23%)	23 (16.43%)	0.59
Oocyte age (years) (3389)	31.83 (6.54)	31.58 (6.98)	0.69
Woman age (years) (3503)	39.84 (5.23)	40.75 (4.71)	0.028
Height (cm) (2829)	164.09 (6.28)	163.81 (5.95)	0.61
Weight (kg) (2741)	63.95 (12.55)	64.67 (13.32)	0.56
BMI (kg/m^2^) (2703)	23.72 (4.51)	23.86 (4.31)	0.72
PGT-a (3503)	802 (23.85%)	32 (22.86%)	0.79
Freeze-all (3503)	2139 (63.60%)	107 (76.43%)	0.002
Endometrial thickness (mm) (3139)	10.29 (2.03)	10.27 (2.61)	0.94
Estradiol level the day before FET (pg/mL) (2238)	224.28 (118.14)	203.64 (112.52)	0.10
Mean serum progesterone levels the day before FET (ng/mL) (2245)	12.53 (6.27)	12.17 (4.20)	0.45
Progesterone < 10.6 ng/mL the day before FET (2245)	875 (40.53%)	35 (40.70%)	0.55
Good quality embryos (A or B, as per the ASEBIR score) (3503)			0.39
0	1100 (32.71%)	50 (35.71%)	
1	2147 (63.84%)	88 (62.86%)	
2	116 (3.45%)	2 (1.43%)	
Mean of transferred embryos (3503)	1.07 (0.21)	1.05 (0.27)	0.12
GnRH agonist pre-treatment (3503)	326 (9.69%)	28 (20%)	<0.001

^1^ Mean (sd) or frequency (%). ^2^ Pearson’s chi-squared test, Fisher’s exact test, Welch two-sample *t*-test.

**Table 2:** Multivariable logistic regression analysis results for clinical pregnancies.

	OR ^1^	95% CI ^1^	*p*-Value
Oocyte age (years)	0.98	0.96, 1.00	0.10
FET			
Embryo donation	-	-	
Oocyte donation	0.85	0.58, 1.25	0.4
Autologous oocyte	1.07	0.69, 1.65	0.8
PGT-a			
No	-	-	
Yes	1.16	0.91, 1.48	0.2
Freeze-all			
No	-	-	
Yes	1.53	1.24, 1.88	<0.001
Progesterone levels the day before FET (ng/mL)			
≥10.6	-	-	
<10.6	0.95	0.80, 1.14	0.6
GnRH agonist pre-treatment	0.93	0.71, 1.21	0.6
No. of transferred embryos	2.36	1.72, 3.28	<0.001
Adenomyosis			
No	-	-	
Yes	0.62	0.39, 0.98	0.04

^1^ aOR—adjusted odds ratio, CI—confidence interval.

**Table 3:** Multivariable logistic regression analysis results for miscarriages.

	OR ^1^	95% CI ^1^	*p*-Value
Oocyte age (years)	1.01	0.98, 1.05	0.6
FET			
Embryo donation	-	-	
Oocyte donation	0.80	0.43, 1.55	0.5
Autologous oocyte	0.86	0.44, 1.76	0.7
PGT-a			
No	-	-	
Yes	0.63	0.41, 0.95	0.031
Freeze-all			
No	-	-	
Yes	0.81	0.56, 1.16	0.2
Progesterone levels the day before FET (ng/mL)			
≥10.6	-	-	
<10.6	1.15	0.85, 1.55	0.4
GnRH agonist pre-treatment	1.33	0.85, 2.03	0.2
No. of transferred embryos	0.74	0.44, 1.17	0.2
Adenomyosis			
No	-	-	
Yes	2.13	0.98, 4.37	0.045

^1^ aOR—adjusted odds ratio, CI—confidence interval.

**Table 4:** Multivariable logistic regression analysis results for live births.

	OR ^1^	95% CI ^1^	*p*-Value
Oocyte age (years)	0.99	0.97, 1.01	0.5
FET			
Embryo donation	-	-	
Oocyte donation	0.90	0.61, 1.35	0.6
Autologous oocyte	0.99	0.64, 1.55	>0.9
PGT-a			
No	-	-	
Yes	1.33	1.05, 1.69	0.017
Freeze-all			
No	-	-	
Yes	1.51	1.22, 1.86	<0.001
Progesterone levels the day before FET (ng/mL)			
≥10.6	-	-	
<10.6	0.93	0.78, 1.11	0.4
GnRH agonist pre-treatment	0.88	0.67, 1.15	0.4
No. of transferred embryos	2.25	1.67, 3.05	<0.001
Adenomyosis			
No	-	-	
Yes	0.46	0.27, 0.75	0.003

^1^ aOR—adjusted odds ratio, CI—confidence interval.

**Table 5:** Multivariable logistic regression analysis results for clinical pregnancies in adenomyosis group.

	OR ^1^	95% CI ^1^	*p*-Value
Oocyte age (years)	0.82	0.69, 0.96	0.016
FET			
Embryo donation	-	-	
Oocyte donation	0.42	0.06, 2.86	0.4
Autologous oocyte	2.85	0.22, 43.5	0.4
PGT-a			
No	-	-	
Yes	2.71	0.76, 10.6	0.13
Freeze-all			
No	-	-	
Yes	4.81	1.33, 21.6	0.024
Progesterone levels the day before FET (ng/mL)			
≥10.6	-	-	
<10.6	0.87	0.30, 2.45	0.8
GnRH agonist pre-treatment	0.68	0.21, 2.13	0.5
No. of transferred embryos	0.56	0.06, 4.60	0.6

^1^ aOR—adjusted odds ratio, CI—confidence interval.

**Table 6:** Multivariable logistic regression analysis results for miscarriages in adenomyosis group.

	OR ^1^	95% CI ^1^	*p*-Value
Oocyte age (years)	1.28	0.89, 2.13	0.2
FET			
Embryo donation	-	-	
Oocyte donation	NA	NA	NA
Autologous oocyte	NA	NA	NA
PGT-a			
No	-	-	
Yes	0.07	0.00, 1.01	0.09
Freeze-all			
No	-	-	
Yes	0.03	0.00, 1.14	0.1
Progesterone levels the day before FET (ng/mL)			
≥10.6	-	-	
<10.6	0.58	0.08, 3.72	0.6
GnRH agonist pre-treatment	0.28	0.01, 2.94	0.3
No. of transferred embryos	0.88	0.01, 64.7	>0.9

^1^ aOR—adjusted odds ratio, CI—confidence interval.

**Table 7:** Multivariable logistic regression analysis results for live births in adenomyosis group.

	OR ^1^	95% CI ^1^	*p*-Value
Oocyte age (years)	0.90	0.75, 1.06	0.2
FET			
Embryo donation	-	-	
Oocyte donation	0.07	0.00, 0.65	0.03
Autologous oocyte	0.26	0.01, 4.84	0.4
PGT-a			
No	-	-	
Yes	2.34	0.63, 9.33	0.2
Freeze-all			
No	-	-	
Yes	9.61	1.48, 203	0.05
Progesterone levels the day before FET (ng/mL)			
≥10.6	-	-	
<10.6	1.35	0.44, 4.13	0.6
GnRH agonist pre-treatment	1.26	0.35, 4.43	0.7
No. of transferred embryos	0.55	0.02, 6.42	0.7

^1^ aOR—adjusted odds ratio, CI—confidence interval.

## Data Availability

Data are available upon request.
